# Thoroughbred Geldings′ Career: Influence of Age at the Start of Training and Racing

**DOI:** 10.3390/ani16040576

**Published:** 2026-02-12

**Authors:** Mailin Hein, Nina Volkmann, Jeanette Probst, Nicole Kemper, Monica Venner

**Affiliations:** 1 Clinic for Horses, University of Veterinary Medicine Hannover, 30559 Hanover, Germany; 2 Institute of Animal Hygiene, Animal Welfare and Farm Animal Behaviour (ITTN), University of Veterinary Medicine Hannover, 30173 Hanover, Germany; 3 Equine Clinic Destedt GmbH, Destedt, 38162 Cremlingen, Germany

**Keywords:** horse, racehorses, Thoroughbreds, two years old, length of career

## Abstract

This retrospective report investigated whether starting racehorses’ training and racing early is associated with a less successful and a shorter racing career. The data of 600 Thoroughbred racehorses from the German Racehorse Association’s archiving programme were evaluated. The horses were classified into three groups regarding their different ages when starting training and racing: early training (16–24 months old)/early racing (two years old); early training/late racing (>two years old); and late training (25–30 months). Statistical models investigated effects on the horses’ length of career. The results showed that the length of their racing career was influenced by sex; geldings showed the longest career as they do not enter breeding programmes, and were analyzed separately. Geldings entering training early, with their first race at two years old, showed higher ratings and a similar length of racing career than those with their first race at three years old or more. Thus, early racing appeared to have had no negative effect on the length of the racing career or perseverance in racing in the geldings investigated. Further studies researching training effects and physiological resilience should consider alternative explanations for racing selection criteria (e.g., early maturation advantage and prior unsoundness) and the reason for ending a racing career.

## Simple Summary

This report describes the length and success of the racing career of German Thoroughbred racehorses with different ages at the start of training and racing, including a special focus on geldings, as their career is not influenced by a breeding perspective. Data from 600 Thoroughbreds were analyzed retrospectively. Horses were classified into three groups concerning their start of training and racing, and variables such as the length of their racing career, performance ratings, their total number of races and lifetime earnings were compared between different groups. The results showed that the horses investigated that started racing early (two years old) had higher performance ratings and a similar length of career than those that raced for the first time when they were older than two years. Especially in geldings, early raced horses achieved higher maximum ratings than those with a later race debut. No negative effect of early training or early racing at two years old on the racing characteristics analyzed was detected. Nevertheless, future prospective studies should include veterinary and management data to evaluate the effect of the individual’s physical and mental state at training and in housing in racing stables, with a final aim of improving the welfare of racehorses.

## 1. Introduction

There has been an ongoing debate since horse racing began regarding the age at which Thoroughbred racehorses should enter training and start in their first race [1,2,3,4]. There is generally a legitimate concern that too-early and inappropriate training is likely, sooner or later, to harm these young horses and cause severe career-ending injuries [5]. These discussions focus on the welfare of the animals, their health and the closely related length of their racing career [6,7]. The Ministry of Agriculture in Germany published guidelines in 2020 that forbid the riding of horses younger than 30 months of age. An exception clause was granted for racehorses for a period of five years [8]. The average age of entry into training in Germany is currently 18 to 24 months, and the first race for two-year-old horses takes place at the end of May each year, which is later than in all other European countries. The German racing rules limit the number of races to a maximum of eight starts at that age [9]. Not all horses start racing within the first year of training. In fact, only thirty-six percent of all two-year-old Thoroughbred racehorses registered as in training in Germany in 2023 had a start in a race.

In this context, one of the questions to address is whether two-year-old Thoroughbred horses have the physical and mental maturity to cope with starting training at the age of 18 months, and to sustain a successful and long career in racing [3,4]. Studies on Thoroughbred racehorses from various countries have shown that starting in races at two years old provides a competitive advantage for these horses throughout their careers [10,11,12,13]. This advantage lies in connection with the musculoskeletal system, which adapts to the increasing training intensity most effectively at an early stage [14,15,16]. If the early training and racing of Thoroughbred racehorses is deleterious, one would expect performance characteristics to be worse in horses trained and raced early. A physical and mental overload would induce more frequent injuries and training interruptions, fewer race starts, lower ratings, lower lifetime earnings and, finally, a shorter racing career. By contrast, several reports have shown greater racing success and a longer racing career in horses that have their first start in racing at two years old compared to those that have their first start at three or four years old [10,11,12,13,17,18,19]. However, to the best of our knowledge, no data have been published on German Thoroughbred racehorses concerning this issue.

The aim of the current report was to describe the age at the start of training and at the first race in relation to the number of races, the lifetime earnings, the rating and the length of a horse’s racing career of German Thoroughbred racehorses. Unlike in other studies, where the duration of the racing career was the time from the first to the last race, we included the start of training in German Thoroughbred horses, as training represents the beginning of physical strain [5]. Additionally, similar to earlier reports, data of all sexes were analyzed. But, in contrast to other studies, where performance characteristics are compared between mares and males, we focused on geldings. This decision was based on the fact that a breeding perspective does not interfere with the planning of racing or bias racing achievements in the group of geldings. Therefore, analyzing geldings separately might provide clearer data on the effect of age at the start in training and racing on the race performance.

## 2. Materials and Methods

### 2.1. Animals and Inclusion Criteria

Data from German Thoroughbred racehorses from several years were analyzed. Specifically, all horses included were born, trained and participated in at least one race in Germany. If the horses were exported abroad in the further course of their career, whether temporarily or permanently, their data was also included in the study if it was available via the ‘Uniturf’ programme described below. According to the German Animal Welfare Law, ethical approval by the research ethics committee was not required due to the retrospective analysis of already documented data.

### 2.2. Data Collection and Processing

German Thoroughbred racehorses born in the years from 2003 to 2005 and from 2011 to 2013 were included in the current study. Two separate periods of time were chosen to compare past and current situations in German horseracing. A hundred horses per year of birth were randomly selected from the ‘General Stud Book, Volume 39.’ The randomization was made by considering the offspring of every seventh mare (prime number) and including it in the study, provided the remaining criteria were met (see above). Data of a total of 600 animals were extracted from the ‘Uniturf’ archiving programme of the German Racehorse Association. These data were originally transmitted into ‘Uniturf’ by breeders, trainers and/or owners and the technical committee department of the German Racehorse Association. Information on the racehorses was entered into Microsoft Excel (version 2016) for data processing with a plausibility check, and was coded for anonymity. All data collected and class descriptions evaluated in this retrospective study are listed in Table 1.

**Figure 1:**
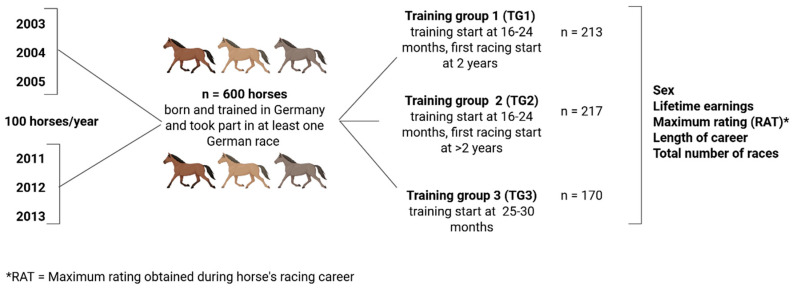
Illustration of the retrospective data analysis showing the number of animals (n), the classification into training groups and the data recorded (Created in BioRender. Kemper, N. (2026) https://BioRender.com/pf5uno1, accessed on 27 January 2026).

In order to evaluate whether the pattern of the number of horses with their first start at two years, three years or an older age differed during the two time periods 2003–2005 and 2011–2013, the data were visually compared using histograms for these six years. There were no differences between the cohorts; therefore, the total population of 600 horses was considered. The animals were divided into three groups according to their age at the beginning of training and their first racing start for further evaluation (Figure 1).

The first training group (TG1; n = 213) included horses that started in training at 16–24 months of age and performed their first race at two years old (early training/early start). The second TG (TG2; n = 217) included horses that started in training at 16–24 months of age and ran their first race at over two years old (early training/late start). The third TG (TG3; n = 170) contained horses that started in training at the age of 25–30 months (late training).

The length of the racing career was defined as the period (in months) between the beginning of training and the last race. The number of horses still racing after the age of seven was very small; therefore, the data of the subsequent years were added to the group ‘seven years and older’. The length of the career was classified into five groups: length of career group 1 (LC1): ≤20 months (n = 112), LC2: >20 and ≤30 months (n = 159), LC3: >30 and ≤40 months (n = 116), LC4: >40 and ≤60 months (n = 110), and LC5: >60 months (n = 103) for further analyses. Finally, the rating (RAT), which is a numerical measure reflecting the quality of a horse’s past performances, was analyzed in 523 horses. Rating is attributed to a horse after its third completed race, and as 77 horses raced only once or twice, these horses had no rating and, thus, were not included in this part of the analysis. Accordingly, the highest rating achieved by each horse was classified into five groups for further statistical processing: RAT1: ≤52.5 (n = 84), RAT2: >52.5 to ≤62.5 (n = 107), RAT3: >62.5 to ≤72.5 (n = 136), RAT4: >72.5 to ≤86.5 (n = 108), and RAT5: >86.5 (n = 88). The groups were selected resulting in consecutive intervals with sample sizes that were as balanced as possible and covered the entire RAT range observed, with the middle range of the distribution being divided into smaller steps, while the peripheral ranges were defined more broadly to ensure sufficient statistical stability with smaller case numbers.

### 2.3. Statistical Analysis

All statistical analyses were carried out using the SAS software (V.9.4, Statistical Analysis Institute, Cary, NC, USA).

A generalized linear mixed model (GLMM) was used to investigate any potential statistical effects on a horse’s length of career given in months. Modelling was performed using a forward selection method with an exclusion criterion set at *p* > 0.05. The model assumptions were checked visually using Q-Q plots for a normal error distribution and the homoscedasticity of residuals.

The model selected as the best-fitting one in data analysis is shown in Equation (1):

*y* = S + RAT + AT + S × RAT + Year/ID (1)

where *y* is the observation on a horse’s length of career given in months; S, RAT and AT are the fixed effects of sex (mare, stallion, or gelding), the grouped racing success (RAT1-RAT5) and the continuous variable of age at the start of training (given in months), respectively. The calculation S × RAT is the fixed effect of (sex × grouped racing success) interaction. Year/ID is the random effect of the horse (ID1-600) nested in the year of birth (2003, 2004, 2005, 2011, 2012 or 2013).

A second GLMM was used to investigate potential effects on the length of career of geldings, where the same modelling procedure as described above was carried out. The best fitting one is shown in Equation (2):

*y* = RAT + AT + Year/ID (2)

where *y* is the observation on gelding’s length of career given in months; RAT and AT are the fixed effects of the grouped racing success (RAT1-RAT5) and the continuous variable of age at the start of training (given in months), respectively. Year/ID is the random effect of the horse (ID1-600) nested in the year of birth (2003, 2004, 2005, 2011, 2012 or 2013).

Post hoc pairwise comparisons of LS means were conducted using Tukey–Kramer tests for both GLMMs.

The variables not included in the generalized linear mixed model were analyzed separately using the Wilcoxon rank-sum test (including an adjustment of *p*-values via Bonferroni–Holm correction) for comparisons of individual data on race performance between the three TGs (TG1–TG3). Furthermore, a chi-square test was used to evaluate differences in proportions of categorical data.

*p*-values of <0.05 were considered statistically significant for all statistical analyses.

## 3. Results

### 3.1. Population’s Age at the Start of Training and Racing Performance

The median age at the start of training of all racehorses recorded (n = 600) was 21.4 months (interquartile range: IQR 19.8–25.7 months) and a total of 9956 race starts were counted (2003: 2013; 2004: 1529; 2005: 1627; 2011: 1546; 2012: 1768; and 2013: 1473). The median lifetime earnings achieved in these races over all horses amounted to 10,485 euros (min: 0; max: 713,220 euros; and IQR: 2400–27,513 euros) and the median earnings per race were 746 euros (min: 0; max: 105,000 euros; and IQR: 286–1870 euros). The median rating determined for all animals analyzed was 68.0 (min: 44.0; max: 102.5; and IQR: 57.5–80.5). The average length of a horse’s racing career recorded overall was 39.0 months (min: 1.0; max: 137.0; and standard deviation: ±24.1 months). The descriptive statistics on the racing performance of the three groups of horses according to their age at the start of training and the first start in racing are listed in Table 2. Statistical comparisons of the lifetime earnings (LE) using the Wilcoxon rank-sum test showed that horses in TG1 earned significantly more than those in TG2 or TG3 (TG1 vs. TG2 and TG3 *p* < 0.0001; TG2 vs. TG3 *p* = 0.015). When comparing the total number of starts in races between the three TGs, the horses of TG1 raced more often than those in TG2 (*p* < 0.0001) and TG3 (*p* = 0.002), while TG2 and TG3 did not differ significantly (*p* = 0.3) (Table 2).

### 3.2. Population’s Length of Racing Career

The mean length of the racing career (LC) of stallions was 38.6 ± 25.1 months; for mares, it was 30.9 ± 17.4 months and for geldings 50.9 ± 27.0 months.

The results of the generalized mixed model showed a relation between the LC and the sex, the grouped racing success, the age at the start of training as well as the interaction (sex × grouped racing success) (Table 3). Pairwise comparisons of LC means between different sexes via the Turkey–Kramer test demonstrated that geldings had a significantly longer career than the other two sexes (both *p* < 0.0001), while the LC between stallions and mares did not differ statistically (*p* = 0.208). Comparing the rating groups, the length of career differed significantly between RAT1 vs. RAT4 (*p* = 0.0129), RAT1 vs. RAT5 (*p* = 0.0003) and RAT3 vs. RAT5 (*p* = 0.0012), showing that the horses with a higher rating had a longer career in Thoroughbred racing.

### 3.3. Geldings’ Age at the Start of Training and Racing Performance

The breeding perspective does not interfere with the planning of racing or influence racing achievements in the group of geldings; therefore, the following analysis focused on geldings. The median age of geldings (n = 204) at the start of training was 21.1 months (min: 16.0; max: 54.0; and IQR 19.5–26.2 months); they started in a median of 19 races (min: 1; max: 108; and IQR: 9–32 races) and achieved median lifetime earnings of 12,828 euros (min: 0; max: 640,272 euros). Their median rating was 64.0 (min: 44.0; max: 97.5; and IQR: 54.0–75.5). Geldings’ total recorded average length of career lasted 50.9 ± 27.0 months (min: 2.4; max: 137.1). The descriptive statistics regarding the racing performance of the geldings within the three TGs are listed in Table 4. The statistical comparisons of the lifetime earnings (LE) using the Wilcoxon rank-sum test showed that the horses in TG1 earned significantly more than those in TG2 (*p* = 0.007) and TG3 (*p* = 0.001), while those in TG2 and TG3 did not differ significantly (*p* = 0.5) (Table 4). When comparing the total number of starts in races between the three TGs, the horses in TG1 raced more often than those in TG2 (*p* = 0.0004) and TG3 (*p* = 0.04). The total number of starts in a race was slightly higher in TG3 than in TG2, without showing a statistically significant difference (*p* = 0.1).

### 3.4. Geldings’ Length of Racing Career

The length of the racing career of geldings in TG1 (early training/early start) was 56.8 ± 27.1 months; this was 52.2 ± 26.5 months in TG2 (late training) and 42.6 ± 26.1 months in TG3 (early training/early start) (Table 4). The results of the GLMM showed a relation between geldings’ LC and the grouped racing success, and the age at the start of training (Table 3). The pairwise comparisons of rating groups showed that geldings’ length of career differed significantly between RAT1 vs. RAT4 (*p* = 0.0005), RAT1 vs. RAT5 (*p* = 0.03), RAT2 vs. RAT4 (*p* = 0.005) and RAT3 vs. RAT4 (*p* = 0.02), showing that geldings with a higher rating also had a longer career in Thoroughbred racing.

Additionally, the perseverance in the racing of geldings of the three TGs was analyzed and the proportions of horses started within a TG were compared using the chi-squared test (Table 5). More geldings in TG1 (early training/early start), at three years old, started in a race than those in both other TGs. The proportion of geldings at seven years old and more in each TG that started in a race was similar.

The number of geldings that started in a race each year from three years old to seven years old and more are shown in Table 6. The age of two years was excluded here, because the majority of geldings that started late in training (TG3) had no start in a race at that age. The proportion of geldings at seven years old and more in each TG (TG1–TG3) that have started each year in a race was similar.

## 4. Discussion

This report describes the association between the age at the start of training, the age at the first start in racing, and the length of the racing career in a cross-cohort population of Thoroughbred racehorses in Germany, retrospectively. The length of the racing career was defined as the duration between the beginning of their training and their last start in a race. This definition was chosen to address the current concerns raised by the German Ministry of Agriculture about the age of racehorses at the beginning of their training [8]. Therefore, we decided to include the training phase, as walking, trotting and slow cantering represent not only a physical effort but also a potentially mental strain [13,20,21,22]. This is supported by data from New Zealand showing that for yearlings and two-year-old horses being prepared for their trial (qualification race), trainers adapt the intensity of training and make decisions to voluntarily interrupt the training of an individual horse if physical or mental development requires to reduce the workload [5].

By contrast, previous studies from Australia, Poland and New Zealand defined the racing career as the time between the first and last racing start [10,11,13]. They reported that horses of all sexes that started racing at two years old had significantly longer racing careers than those that started their career at three years old [10,11,13]. Despite the difference in the definition of the length of the career, the current report also showed that the group of horses beginning in training at the age of 16 to 24 months had longer careers over all sexes than the horses with a late start of training (TG3).

The statistical analyses revealed a significant effect of sex on the length of the racing career. The geldings’ career was significantly longer than those of mares or stallions. The racing career of most mares and stallions probably ended shortly after proving their breeding value. In those cases, they are transferred in a timely manner to breeding programmes [23,24]. Under these conditions, the length of racing career of geldings might not reflect primarily physiological resilience, soundness or training impact, but alternative career pathways. However, an answer to the question regarding whether early training and racing are detrimental to racehorses based on the retrospective data can only be found by evaluating geldings, as breeding does not interfere with their racing characteristics. Previous studies showed that the career of Thoroughbred racehorses was influenced to varying degrees by different factors. In addition to sex, factors such as training management, race planning, athletic ability, genetic and epigenetic characteristics as well as the race earnings of the individual horses were mentioned [23,25,26,27,28]. Even though no other authors have investigated the rating previously, we analyzed this performance characteristic as a possible influencing factor affecting the length of a horse’s racing career. It is common that the evaluation of the athletic performance of a racehorse is based on the highest rating of the career. The rating is an independent performance parameter, as it is based mainly on the position of the horse at the end of a race and awarded according to the same criteria in every German race [9]. The length of the racing career in horses of all sexes increased with increasing ratings in the current study, which conversely means that horses with a lower rating had a shorter career in active racing. This influence of racing success or a lack of ability as a racehorse on the length of the racing career in Germany corresponds to statements in previous studies from countries such as Australia, New Zealand and Poland [10,11,12,13].

Success in racing is an essential breeding selection trait, which is listed in the breeding programme of the German Racehorse Association [9,23] and in many other countries [10,11,12]. However, previous studies from other countries only differentiated between males and female horses. In the current report, stallions, mares and geldings were considered separately when investigating the key question of “the age at the start of training and the length of the racing career” to avoid the significant influence of the breeding aspect. Therefore, geldings’ data were analyzed separately, as they race for a longer time, because they do not enter breeding programme. Nonetheless, the results regarding the geldings showed that those starting training at the age of 16–24 months and racing at the age of two years (TG1) had a similar length of racing career (56.8 ± 27.1 months) compared to those with an early start of training and race debut at three years of age (TG2; 52.2 ± 26.5 months). Furthermore, geldings with an early start of training and racing (TG1) had a significantly higher rating and lifetime earnings than those that started in a race at an older age (TG2). These results suggest that an early start in racing has no negative influence on the length of the career or racing performance of geldings. This contrasts with concerns that starting a race at two years old is associated with high training intensity and a physical and mental overload. One might expect an early start in a race to result in more injuries, training breaks and fewer horses performing in races after the age of six. Our results showed no reduction in the racing performance, shortened racing careers or fewer starters at six years old or older in the investigated horses. This finding was similar to that of studies from other countries, despite other criteria relating to the sex and length of the racing career. Additionally, in previous studies, horses that had their first start at two years old had longer racing careers and higher lifetime earnings than those with their first start in a race at three or four years old [17,29,30]. However, data on the age at the start of training are lacking in those studies [10,11,12]. In the present report, 42.2% of geldings trained at an early age started competing at the age of two. It would therefore be particularly interesting to investigate why the remaining 57.8% of early trained geldings had their racing debut at the age of three or older.

One limitation of the current report is that the horses of the three groups compared here (different age at the start of training and first start) were not evenly allocated to each group. It cannot be excluded that different reasonings behind a later start of training and racing, such as the decisions of the owner mainly based on the pedigree of the horse or body development, played a role, but these were not recorded. Therefore, the group of horses that were trained and raced late might have had a maturity disadvantage at the age of 16–24 months compared to the others. This potential bias can only be ruled out in a prospective study with a randomized allocation of horses of different genetics and development in the three training groups. A further limitation is related to the horses that were exported, as this data collection was dependent on the submission by trainers and owners, as the report of this information is not mandatory. In those few cases, lifetime earnings might be reduced and the length of career shortened. Additionally, the race system forces trainers to make strategic nominations that do not always lead to maximum performance and earnings from each horse. Finally, the entry criteria of races and the relatively small number of races in Germany also limit the options of adequate races and, consequently, the ability of each horse to perform at maximum capacity.

Previous studies have stated that the management of training, planning of races, health, athletic ability, and genetics and epigenetics of the horse are also essential factors affecting the course of each horse′s racing career and its length [24,25,27,28]. Additionally, the trainer and owner’s decisions are very individual, subjective and not comparable, but might have a major influence on the racing performance (including duration and success) as well as health and welfare of racehorses. Thus, in the present report, it is likely that the group of horses trained early and started at age of two represent a previous selection of physiologically high-performance individuals. Therefore, several authors faced difficulties in analyzing these factors in other countries [10,11,12,13,20,21,22,31,32,33,34,35]. The analysis of data of young Thoroughbred racehorses in preparation for the trial in New Zealand showed that trainers make individual decisions regarding the interruption of training based, firstly, on the development and behaviour of the horses and based, secondly, on health issues such as infectious disease or injuries. Especially, high-speed training was associated with more frequent training interruptions and a later start in racing [5]. This shows that training regimens should be well monitored and adapted to each individual horse.

Individual trainer data should be included in future studies to improve the analysis of these factors. The reasons why horses are temporarily or definitively taken out of training should also be analyzed in order to address the welfare of racehorses more precisely. Furthermore, health parameters, for example, injuries, gastric ulcers or other possible restrictive incidents, should be considered in a prospective study to provide an objective statement regarding the influence of age at the start of training and the age of the first race on the career of German Thoroughbred racehorses.

## 5. Conclusions

Thoroughbred racehorses that started training at the age of 16 to 24 months and that had their first race at two years old had better performance parameters (rating and total number of starts) than those that participated in their first race at three years old. Specifically, in the investigated geldings, starting training at the age of 16 to 24 months and participating in their first race at two years old appeared to have no negative effect on the length of the racing career and perseverance in racing compared to geldings that participated in their first race at three years old. As not all early trained horses also raced at the age of two years, care should be taken in making tailored decisions regarding the training programme and intensity for each individual horse based on daily physical evaluation and behaviour.

## Figures and Tables

**Table 1:** Basic data chosen of all Thoroughbred horses included.

Variable	Explanations and Definition
Horses ID	Horses were coded for anonymity with an identification number (ID from 1 to 600)
Sex	Stallion, mare, or gelding (geldings = males castrated before the end of their racing career)
Age at the start of training (AT) (months)	Age at which horses were put on an official training list
Total number of races (TR)	Number of domestic and foreign starts (flat track/obstacle races included)
Lifetime earnings (LE) (euros)	Earnings from domestic and foreign racing starts (flat track/obstacle races combined)
Length of career (LC) (months) Rating (RAT)	Time from the beginning of training to the last race Maximum rating obtained during the horse’s racing career

**Table 2:** Descriptive statistics on the racing performance of the different groups of horses over all sexes according to their age at the start of training and their first racing start (TG1: early training/early start; TG2: early training/late start; and TG3: late training).

Variable	Median	Min	Max	IQR
TG1 (n = 213): 43 stallions, 108 mares, and 62 geldings				
AT (months)	20.5	16.1	24.4	19.2–21.5
LE (euros)	18,170	0	525,000	8222–47,050
Rating (RAT; n = 201)	74.5	46.5	102.5	67.0–88.0
LC * (months)	42.0	7.8	137.1	±24.4
TR (n)	14	1	106	8–25
TG2 (n = 217): 35 stallions, 97 mares, and 85 geldings				
AT (months)	20.6	16.5	24.4	19.5–21.6
LE (euros)	8200	0	713,220	1600–25,400
Rating (RAT; n = 179)	65.5	44.0	98.5	57–76.5
LC * (months)	42.2	15.1	134.3	±23.4
TR (n)	9	1	102	4–20
TG3 (n = 170): 20 stallions, 93 mares, and 57 geldings				
AT (months)	31.9	24.5	58.2	27.4–36.4
LE (euros)	5450	0	361,540	570–13,600
Rating (RAT; n = 179)	58.0	44.0	96.0	50.5–68.5
LC * (months)	31.2	1.3	115.3	±23
TR (n)	9	1	108	5–22

* Normally distributed variable: mean and standard deviation is indicated. AT = age at the start of training; LE = lifetime earnings; RAT = rating; LC = length of career; TR = total number of starts in races; min = minimum; max = maximum; and IQR = interquartile range.

**Table 3:** Results of the generalized mixed models (GLMM) calculated for population’s and geldings’ length of career (LC), showing the number of degrees of freedom (Num DF), and the calculated F- and *p*-values.

GLMM on Populations’ LC	Effect	Num DF	F-Value	*p*-Value
	Age at the start of training (months)	33	1.60	0.020
	Grouped racing success	4	4.32	0.0019
	Sex	2	46.08	<0.0001
	Sex × grouped racing success	8	4.69	<0.0001
GLMM on geldings’ LC	Age at the start of training (months)	30	1.13	0.030
	Grouped racing success	4	3.93	0.004

**Table 4:** Descriptive statistics on the racing performance of the different groups of geldings according to their age at the start of training and their first racing start (TG1: early training/early start; TG2: early training/late start; and TG3: late training).

Variable	Median	Min	Max	IQR
TG1 (n = 62)				
AT (months)	20.0	16.1	24.3	18.7–21.1
LE (euros)	19,505	0	329,425	10,470–41,520
Rating (RAT; n = 62)	72.0	46.5	96.0	63.0–78.0
LC * (months)	56.8	17.5	137.1	±27.1
TR (n)	24	4	106	12–39
TG2 (n = 85)				
AT (months)	20.4	16.5	24.4	19.1–21.5
LE (euros)	10,200	0	640,272	1200–29,150
Rating (RAT; n = 72)	63.8	44.0	97.5	55.8–74.8
LC * (months)	52.2	15.1	134.3	±26.5
TR (n)	15	1	102	5–25
TG3 (n = 57)				
AT (months)	31.8	24.5	54.0	28.2–36.4
LE (euros)	11,100	0	219,200	1600–18,250
Rating (RAT; n = 52)	55.8	44.0	96.0	50.0–64.3
LC * (months)	42.6	2.4	115.3	±26.1
TR (n)	22	2	108	8–29

* Normally distributed variable: mean and standard deviation is indicated. AT = age at start of training; LE = lifetime earnings; RAT = rating; LC = length of career; TR = total number of starts in races, min = minimum; max = maximum; and IQR = interquartile range.

**Table 5:** Number of geldings of the three TGs (TG1: early training/early start; TG2: early training/late start; and TG3: late training) that started in a race at the stated age.

Training Group	n (Total = 204)	Started at 2 Years Old (n)	Started at 3 Years Old (n)	Started at 4 Years Old (n)	Started at 5 Years Old (n)	Started at 6 Years Old (n)	Started at 7 Years Old and Older (n)
TG1	62	62 ^a^ (100%)	60 ^a^ (96.8%)	55 ^a^ (88.7%)	40 ^a^ (64.5%)	29 ^a^ (46.8%)	22 ^a^ (35.5%)
TG2	85	0	66 ^b^ (77.6%)	72 ^a^ (84.7%)	40 ^b^ (47.1%)	35 ^a^ (41.2%)	28 ^a^ (32.9%)
TG3	57	5 ^b^ (8.8%)	40 ^b^ (70.2%)	50 ^a^ (87.7%)	37 ^a^ (64.9%)	30 ^a^ (52.6%)	17 ^a^ (29.8%)

^a,b^ = Proportions of geldings that started were analyzed using the chi-square test; numbers in the same column with dissimilar superscripts differed significantly (*p* < 0.05).

**Table 6:** Number of geldings of the three TGs (TG1: early training/early start; TG2: early training/late start; and TG3: late training) that started in a race in each year from three to seven years old and older.

Training Group	n (Total = 204)	Started at 3 Years Old (n)	Started at 4 Years Old (n)	Started at 5 Years Old (n)	Started at 6 Years Old (n)	Started at 7 Years Old and Older (n)
TG1	62	60 ^a^ (96.8%)	53 ^a^ (85.5%)	38 ^a^ (61.3%)	25 ^a^ (40.3%)	19 ^a^ (30.6%)
TG2	85	66 ^b^ (77.6%)	56 ^b^ (65.9%)	31 ^b^ (36.5%)	25 ^a^ (29.4%)	16 ^a^ (18.8%)
TG3	57	40 ^b^ (70.2%)	34 ^b^ (59.6%)	23 ^b^ (40.4%)	19 ^a^ (33.3%)	11 ^a^ (19.3%)

^a,b^ = Proportions of geldings that started were analyzed using the chi-square test; numbers in the same column with dissimilar superscripts differed significantly (*p* < 0.05).

## Data Availability

The original contributions presented in this study are included in the article. Further inquiries can be directed to the corresponding author.
